# A novel method for genomic-enabled prediction of cultivars in new environments

**DOI:** 10.3389/fpls.2023.1218151

**Published:** 2023-07-25

**Authors:** Osval A. Montesinos-López, Sofia Ramos-Pulido, Carlos Moisés Hernández-Suárez, Brandon Alejandro Mosqueda González, Felícitas Alejandra Valladares-Anguiano, Paolo Vitale, Abelardo Montesinos-López, José Crossa

**Affiliations:** ^1^ Facultad de Telemática, Universidad de Colima, Colima, Colima, Mexico; ^2^ Centro Universitario de Ciencias Exactas e Ingenierías (CUCEI), Universidad de Guadalajara, Guadalajara, Jalisco, Mexico; ^3^ Instituto de Innovaciónn y Desarrollo, Universidad Francisco Gavidia, San Salvador, El Salvador; ^4^ Centro de Investigación en Computación (CIC), Instituto Politécnico Nacional (IPN), México City, Mexico; ^5^ Centro de Investigación y Formación del Pensamiento Libre en México, Colima, Mexico; ^6^ International Maize and Wheat Improvement Center (CIMMYT), El Batan, Edo. de México, Mexico; ^7^ Colegio de Postgraduados, Montecillos, Edo. de México, Mexico; ^8^ Centre for Crop & Food Innovation, Food Futures Institute, Murdoch University, Perth, WA, Australia

**Keywords:** gains in accuracy, GBLUP, genomic prediction, genotype × environment interaction (GE), novel method

## Abstract

**Introduction:**

Genomic selection (GS) has gained global importance due to its potential to accelerate genetic progress and improve the efficiency of breeding programs.

**Objectives of the research:**

In this research we proposed a method to improve the prediction accuracy of tested lines in new (untested) environments.

**Method-1:**

The new method trained the model with a modified response variable (a difference of response variables) that decreases the lack of a non-stationary distribution between the training and testing and improved the prediction accuracy.

**Comparing new and conventional method:**

We compared the prediction accuracy of the conventional genomic best linear unbiased prediction (GBLUP) model (M1) including (or not) genotype × environment interaction (GE) (M1_GE; M1_NO_GE) versus the proposed method (M2) on several data sets.

**Results and discussion:**

The gain in prediction accuracy of M2, versus M1_GE, M1_NO_GE in terms of Pearson´s correlation was of at least 4.3%, while in terms of percentage of top-yielding lines captured when was selected the 10% (Best10) and 20% (Best20) of lines was at least of 19.5%, while in terms of Normalized Root Mean Squared Error (NRMSE) was of at least of 42.29%.

## Introduction

Genomic selection (GS) has gained global importance due to its potential to revolutionize agriculture by accelerating genetic progress and improving the efficiency of breeding programs (Heffner et al., 2009). The increasing global population also increases the demand for food, making it necessary to improve agricultural productivity and sustainability. GS optimizes and helps achieve these goals by enabling breeders to select individuals with superior genotypes that exhibit desirable traits, such as high yield, disease resistance, and environmental adaptability. This methodology has been successfully applied in various crops, such as maize, wheat, and rice, as well as in livestock breeding, such as dairy cattle, beef cattle, and pigs (VanRaden et al., 2009). By harnessing the power of genomics, breeders can identify and utilize favorable genetic variants that would otherwise be difficult to identify with traditional breeding methods. This enhances the efficiency and accuracy of selecting superior individuals while reducing the need for costly and time-consuming field testing. As such, GS has become an important tool that contributes to a more sustainable and resilient agriculture and meets the demands of a growing global population (Daetwyler et al., 2013).

Nevertheless, GS is challenging when predicting the performance of lines in new environments due to a phenomenon called genotype-by-environment interaction (G× E). G×E occurs when the effect of a genotype on a trait differs across different environments, such as different geographic locations or weather conditions (Gianola and Rosa, 2015; Crossa et al., 2017; Resende et al., 2018). Since genomic prediction models are typically developed using data from specific environments, the models may not accurately predict the performance of lines in other environments. For this reason, it is important to account for the G×E effect, as it can result in a significant decrease in prediction accuracy when ignored (Crossa et al., 2017; Resende et al., 2018). To address this challenge, breeders collect data from multiple environments to develop more robust models that can account for the G×E effect. They also use prediction models that model G×E to identify genomic regions that exhibit stable performance across different environments. By considering the G×E effect, breeders can develop more accurate genomic prediction models that can be used to predict the performance of lines in new environments (Crossa et al., 2017; Resende et al., 2018).

Several genomic prediction approaches can be used to improve the prediction of lines in new environments. One approach is to collect data from multiple environments and use it to develop more robust genomic prediction models that can account for the (G×E) effect (Hickey and Gorjanc, 2020). Another approach is to use machine learning algorithms that can identify important environmental variables that contribute to the G×E effect and incorporate them into the genomic prediction models (Guo et al., 2019). Additionally, researchers can develop multi-trait models that predict multiple traits simultaneously, as this can improve the accuracy of prediction across environments. Yet another approach is to use a multi-population model, which incorporates data from multiple populations with different levels of relatedness to increase the diversity of the training population. Finally, incorporating functional genomic data, such as gene expression or epigenetic data can also improve the prediction accuracy in new environments. Overall, a combination of these approaches may be required to address the challenge of predicting the performance of lines in new environments.

Most statistical and machine learning models can fail to accurately predict performance in a completely new environment because they rely on assumptions and patterns learned from the data on which they were trained, and these may not hold true in a new environment or dataset that has different underlying characteristics (Hastie et al., 2009). Also, predicting specific genotypes in new environments becomes challenging when faced with such high levels of genetic variability and the environments used as training and testing are very different.

In statistical models, the assumptions made about the distribution of the data, the relationships between variables and the model structure may differ in a new environment. For example, if a linear regression model is trained on data that has a linear relationship between the independent and dependent variables, and in a new environment the relationship is non-linear, the model will not perform well in making predictions (Breiman, 2001).

Similarly, machine learning models can overfit the training data, which means that they may learn the noise or idiosyncrasies of the specific training data rather than the general patterns that can be applied to new data (Goodfellow et al., 2016). This can result in poor performance when predicting in a new environment with different data. To address this difficulty, there are two approaches that can be considered. The first approach involves having access to diverse and representative training data and using conventional models and techniques, such as cross-validation, to improve the prediction of genotypes in new environments (Hastie et al., 2009). The second approach is to propose radical modeling approaches that can significantly enhance the prediction of tested genotypes in new environments compared to conventional models. Since achieving high prediction accuracy for tested genotypes in completely new environments is very challenging, this paper explores a novel method with a fundamentally different modeling approach from conventional models. Instead of directly training the prediction models with the response variable, as typically done in conventional models, the proposed model is trained using the difference of response variables between environments in the training set. This approach is crucial for reducing the distribution mismatch between the training and testing sets. Additionally, it allows for the computation of predictions for the tested genotypes in the new environment as an ensemble. The final predictions are generated by combining information from the response variables of each environment in the training set and predictions of the multiple differences. The number of differences computed is denoted by $\left(\begin{array}{c} I\\ 2 \end{array}\right)$ , where I represent the number of environments available in the training set.

## Materials and methods

### Data sets

We present the eight data sets used for benchmarking the proposed method and the conventional methods. These datasets have been employed in previous studies and the quality control for the genomic data consists in removing markers that had more than 15% missing values and those with a minor allele frequency (MAF) of< 0.05.

#### Data set 1. Maize

This maize data set was included in Souza et al. (2017) and comes from the University of Sao Paulo (USP). It consists of 722 (with 722 × 4 = 2888 observations) maize hybrids obtained by crossing 49 inbred lines. The hybrids were evaluated in four environments (E1-E4) in Piracicaba and Anhumas, São Paulo, Brazil, in 2016 using an augmented block design with two commercial hybrids as checks to correct for micro-environmental variation. The parent lines were genotyped with an Affymetrix Axiom Maize Genotyping Array of 616 K SNPs. Markers with MAF of 0.05 were removed. After applying QC, 54,113 SNPs were available to make the predictions.

#### Data set 2. Japonica

Monteverde et al. (2019) reported a rice data set that belongs to the tropical Japonica population with four traits (namely Grain Yield (GY): Percentage of Head Rice Recovery (PHR), Percentage of Chalky Grain (GC), and Plant Height (PH)) and in four environments (years: 2010, 2011, 2012, and 2013). A total of 127 lines were evaluated each year, and 16,383 SNP markers remained after coding the marker as 0, 1 and 2. In this vein, a total of 508 assessments were evaluated in the four environments.

#### Data set 3 (EYT_1), data set 4 (EYT_2), and data set 5 (EYT_3)

These three data sets were collected by the Global Wheat Program of the International Maize and Wheat Improvement Center (CIMMYT) and belong to elite yield trials (EYT) established in four different cropping seasons with four environments. Data set 3 (EYT_1) is from cycle 2013–2014; data set 4 (EYT_2) is from cycle 2014–2015; and data set 5 (EYT_3) is from cycle 2015–2016. The EYT data sets 1, 2 and 3 contain 766, 775 and 964 lines, respectively.

An alpha-lattice design was used as the experimental design, and the lines were sown in 39 trials, each covering 28 lines and two checks in six blocks with three replications. Several lines and traits were available for some environments in each data set. In this study, we included four traits measured for each line in each environment: days to heading (DTHD; number of days from germination to 50% spike emergence); days to maturity (DTMT, number of days from germination to 50% physiological maturity or the loss of the green color in 50% of the spikes); PH; and GY. For full details on the experimental design and how the best linear unbiased estimates (BLUEs) were computed, see Juliana et al. (2018).

The lines examined in the data sets were evaluated in four environments. For data set 3 (EYT_1), the environments were bed planting with five irrigations (Bed5IR), early heat (EHT), flat planting and five irrigations (Flat5IR), and late heat (LHT). For data set 4 (EYT_2), the environments were bed planting with two irrigations (Bed2IR), Bed5IR, EHT, and Flat5IR. For data set 5 (EYT_3), the environments evaluated were Bed2IR, Bed5IR, Flat5IR, and flat planting with drip irrigation (FlatDrip). Genome-wide markers for the 2515 (776 + 775 + 964) lines in the three data sets were obtained through genotyping-by-sequencing (Elshire et al., 2011) at Kansas State University using an Illumina HiSeq2500. After filtering, 2038 markers were obtained from an initial set of 34,900 markers. The imputation of missing markers data was carried out using LinkImpute and implemented in TASSEL (Trait Analysis by Association Evolution and Linkage) version 5 (Bradbury et al., 2007). Lines that had more than 50% missing data were removed, providing a final total of 2515 lines that were used in this study (776 lines in the third data set, 775 lines in the fourth data set, and 964 lines in the fifth data set).

#### Data set 6. Indica

This dataset contains information on the phenotypic performance of the same four traits reported for rice from the Japonica data set (GY, PHR, GC and PH), which was also reported also by Monteverde et al. (2019) in three environments in 2010, 2011 and 2012. For each year, 327 lines were evaluated. The total number of assessments in this balanced data set is 981 since each line is included once in each environment. After quality control, markers for 16,383 SNPs remained and were coded as 0, 1, and 2.

#### Data set 7. Groundnut

This data set was reported by Pandey et al. (2020) and includes information on the phenotypic performance of 318 groundnut lines for various traits in four locations. The traits under study include pods per plant (NPP), pod yield per plant (PYPP) measured in grams, seed yield per plant (SYPP) measured in grams and yield per hectare (YPH) measured in kilograms. The four locations are denoted as Aliyarnagar_R15, Jalgoan_R15, ICRISAT_R2015, and ICRISAT_PR15-16. The data set is balanced, giving a total of 1272 assessments with each line included once in each location. Marker data were available for all lines, and 8268 single-nucleotide polymorphism (SNP) markers remained after quality control (with each marker coded with 0, 1, or 2).

#### Data set 8. Disease

In this data set with 438 wheat lines, three traits (diseases) were measured: *Pyrenophora tritici-repentis*, which causes a disease known as yellow spot, yellow leaf spot, tan spot, yellow leaf blotch, or helminthosporiosis; *Parastagonospora nodorum*, a major wheat fungal pathogen that affects the leaves and other parts of the plants; and *Bipolaris sorokiniana*, the cause of seedling diseases, common root rot and spot blotch in several crops such as barley and wheat. Over a long period during the same year, these 438 lines were evaluated in the greenhouse for several replicates, which were subsequently considered environments (Env1, Env2, Env3, Env4, Env5, and Env6) as the experiments were established in different greenhouses and planting dates. For the three traits measured, the total number of observations was 438×6=2628 . It should be noted that this data set was also used by Montesinos-López et al. (2020). The response was measured on a continuous scale from 1 (resistance) to 5 (susceptible).

DNA samples were genotyped using 67,436 SNPs. For a given marker, the genotype for each line was coded as the number of copies of a designated marker-specific allele carried by the line (absence = zero and presence = one). The markers that had more than 15% missing values were removed, as well as markers with MAF< 0.05. A total of 11,617 SNPs were still available for analysis after quality control and imputation.

### M1-Conventional GBLUP model

One of the predictors implemented under a GBLUP model was:


(1) $$Y_{ij}=\mu +L_{i}+g_{j}+gL_{ij}+\varepsilon_{ij}$$

where $Y_{ij}$ denotes the response variable measured at genotype j at environment i; μ denotes a general mean; $L_{i}$ denotes the random effects of environments (or locations) distributed as $\text{L}={\left(L_{1},\ldots ,L_{I}\right)}^{T}∼N_{ℐ}\left(0,\sigma_{L}^{2}\text{H}\right)$ , where H denotes the environmental relationship matrix computed as proposed by VanRaden (2008), but in place of using genomic information, it was estimated using only the design matrix of environments; that is, $\text{H}=\frac{X_{L}X_{L}^{T}}{r}$ , where ${\text{X}}_{L}$ is the design matrix of environments of dimension n×I , where n denotes the number of observations; $g_{j},j=1,\ldots ,J$ , are the random effects of lines, $gL_{ij}$ are the random effects of environment × line interaction (GE) and $\epsilon_{ij}$ denotes the random error terms assumed normally distributed with mean 0 and variance $\sigma^{2}$ . Furthermore, it is assumed that $\text{g}={\left(g_{1},\ldots ,g_{J}\right)}^{T}∼N_{J}\left(0,\sigma_{g}^{2}\text{G}\right)$ , $\text{gL}={\left(gL_{11},\ldots ,gL_{1J},\ldots ,gL_{IJ}\right)}^{T}∼N_{ℐJ}\left(0,\sigma_{gL}^{2}\text{H}⊙Z_{g}\text{G}Z_{g}^{T}\right)$ , where G is the genomic relationship matrix (VanRaden, 2008), ⊙ denotes the Hadamard product and H is the environment relationship matrix of size nxn . When Model (1) was implemented, it was denoted as M1_GE, but when model (1) was implemented without the genotype by environment interaction component ( $gL_{ij}$ ) it was denoted as M1_NO_GE. The implementation of these models was done in the R statistical software (R Core Team, 2023) version 4.2.3 in the BGLR library of Pérez and de los Campos, 2014.

### M2-Proposed method

This method is proposed for the prediction of tested lines in untested environments. We assume that we have a balanced data set where the same J lines were evaluated in all the environments (E) and the number of environments is at least I=3. Assuming that we have only one response variable ( $y_{ij}$ for i=1,.,I, j=1,.,J). The method consists of the following steps:

**Step 1**: Compute all resulting combination (RC) of choosing two environments of all available environments (I), that is, $RC=\left(\begin{array}{c} I\\ 2 \end{array}\right)$ .

**Step 2**: Make explicit all the combinations of two environments: ( $E_{1},E_{2}$ ) … ( $E_{1},E_{I}$ )…( $E_{I-1},E_{I}$ ).

**Step 3**: Compute the variable difference ( $d_{i,i^{\prime },j}=y_{ij}-y_{i^{\prime }j}$ ) for each pair of environments.

**Step 4**: Build a data frame by stacking all the RC blocks of variable difference ( $d_{i,i^{\prime },j}=y_{ij}-y_{i^{\prime }j}$ ) computed in Step 3. Also we add others columns of the same length to identified lines, Env_i, Env_i´, yij, yi´j, and $d_{i,i^{\prime },j}$ .

**Step 5**. Since the goal of prediction is tested lines in untested environments, we put NA in the new response variable, $d_{i,i^{\prime },j}$ , where is present the environments that will be predicted. Then all rows of this data frame with NAs conform the testing set and the rows without NAs in $d_{i,i^{\prime },j}$ correspond the training set. Then we train the following GBLUP model:


(2) $$d_{i,i^{\prime },j}=\mu +g_{j}+\varepsilon_{ij}$$

Where μ is also a general intercept, $g_{j}$ is the random effect of genotypes with the same distribution as given in model (1), $\varepsilon_{ij}$ is a residual term with normal distribution and mean 0 and variance $\sigma^{2}$ . However, now the response variable is $d_{i,i^{\prime },j},$ instead of the original response variable ( $y_{i,j}$ ). It is also important to point out that now with model (2) we made predictions for the observations of the variable difference of the testing set ( ${\hat{d}}_{i,i^{\prime },j}$ ), not for the original response variable. For this reason, the final predictions of the testing set in terms of the original response variables are obtained as a kind of ensemble with the following expression:


(3) $${\hat{y}}_{i,j}=\frac{1}{I-1}\sum_{i^{\prime }=1}(y_{i^{\prime },j}+{\hat{d}}_{i,i^{\prime },j})$$

Next, to clarify the steps required for implementing the proposed method M2, we provide an example for J=3 lines and I=3 environments, and we denote environment 1as $E_{1}$ , environment 2 as $E_{2}$ and environment 3 as $E_{3}$ . For Step 1, we compute $RC=\left(\begin{array}{c} 3\\ 2 \end{array}\right)=3$ . For Step 2, the following are the resulting combinations: ( $E_{1},E_{2}$ ), ( $E_{1},E_{3}$ ) and ( $E_{2},E_{3}$ ). For Step 3 these are the following variable differences:


$${\text{d}}_{1,2}=\left(y_{11}-y_{21},y_{12}-y_{22},y_{13}-y_{23}\right),$$

$${\text{d}}_{1,3}=\left(y_{11}-y_{31},y_{12}-y_{32},y_{13}-y_{33}\right),$$

$${\text{d}}_{2,3}=\left(y_{21}-y_{31},y_{22}-y_{32},y_{23}-y_{33}\right)$$

For Step 4 we provide the following table (**Table 1**) that will be the input for the data.frame.

**Table 1:** Modified input for implementing model M2 with I=3 environments and J=3 environments.

Obs.	*Line*	Env_i	Env_i´	*y_ij_*	$y_{i´j}$	$d_{i,i´,j}$
1	*g*_1_	*E*_1_	*E*_2_	*y*_11_	*y*_21_	*y*_11_ *− y*_21_
2	*g*_2_	*E*_1_	*E*_2_	*y*_12_	*y*_22_	*y*_12_ *− y*_22_
3	*g*_3_	*E*_1_	*E*_2_	*y*_13_	*y*_23_	*y*_13_ *− y*_23_
4	*g*_1_	*E*_1_	*E*_3_	*y*_21_	*y*_31_	*y*_11_ *− y*_31_
5	*g*_2_	*E*_1_	*E*_3_	*y*_22_	*y*_32_	*y*_12_ *− y*_32_
6	*g*_3_	*E*_1_	*E*_3_	*y*_23_	*y*_33_	*y*_13_ *− y*_33_
7	*g*_1_	*E*_2_	*E*_3_	*y*_31_	*y*_11_	*y*_21_ *– y*_31_
8	*g*_2_	*E*_2_	*E*_3_	*y*_32_	*y*_12_	*y*_22_ *− y*_32_
9	*g*_3_	*E*_2_	*E*_3_	*y*_33_	*y*_13_	*y*_23_ *− y*_33_

For Step 5, we assume that $E_{1}$ will be the testing set while environments $E_{2}$ and $E_{3}$ the training set. For this reason, the final input for training is given in **Table 2**.

**Table 2:** Modified input for implementing model M2 with I=3 environments and J=3 environments, with NAs in the column $d_{i,i´,j}$ for testing set and the remaining for the training set.

Obs.	*Line*	Env_i	Env_i´	*y_ij_*	$y_{i´j}$	$d_{i,i´,j}$
1	*g*_1_	*E*_1_	*E*_2_	*y*_11_	*y*_21_	NA
2	*g*_2_	*E*_1_	*E*_2_	*y*_12_	*y*_22_	NA
3	*g*_3_	*E*_1_	*E*_2_	*y*_13_	*y*_23_	NA
4	*g*_1_	*E*_1_	*E*_3_	*y*_21_	*y*_31_	NA
5	*g*_2_	*E*_1_	*E*_3_	*y*_22_	*y*_32_	NA
6	*g*_3_	*E*_1_	*E*_3_	*y*_23_	*y*_33_	NA
7	*g*_1_	*E*_2_	*E*_3_	*y*_31_	*y*_11_	*y*_21_ *− y*_31_
8	*g*_2_	*E*_2_	*E*_3_	*y*_32_	*y*_12_	*y*_22_ *− y*_32_
9	*g*_3_	*E*_2_	*E*_3_	*y*_33_	*y*_13_	*y*_23_ *− y*_33_

Now the predicted values were for: ${\text{d}}_{1,2}=\left(y_{11}-y_{21},y_{12}-y_{22},y_{13}-y_{23}\right)$ and ${\text{d}}_{1,3}=\left(y_{11}-y_{31},y_{12}-y_{32},y_{13}-y_{33}\right)$ . For this reason, the final prediction of each line in the testing set, $E_{1},$ in terms of the original response variables can be obtained as:


$${\hat{y}}_{1,1}=\frac{1}{2-1}[(y_{2,1}+{\hat{d}}_{1,2,1})+(y_{3,1}+{\hat{d}}_{1,3,1})],$$

$${\hat{y}}_{1,2}=\frac{1}{2-1}[(y_{2,2}+{\hat{d}}_{1,2,2})+(y_{3,2}+{\hat{d}}_{1,3,2})],$$

$${\hat{y}}_{1,3}=\frac{1}{2-1}[(y_{2,3}+{\hat{d}}_{1,2,3})+(y_{3,3}+{\hat{d}}_{1,3,3})]$$

### Cross-validation strategy and evaluation metrics

The cross-validation strategy used was to leave one environment out. Under this strategy at each iteration, all data of an environment is used as the testing set and the remaining data from the rest of environments is used as the training set (Montesinos López et al., 2022). There are as many iterations as environments so that each environment can be used at least once as the testing. Using this method we sought to assess how well a model can predict information of a complete environment using data from other, different environments. To evaluate the prediction accuracy of the proposed method (M2) versus the conventional methods (M1_GE and M1_NO_GE), we employed the following three metrics: (1) normalize root mean square error, (2) Average Pearson’s correlation (APC) computed between the observed and predicted values of the testing set. (3) Percentage of top-yielding lines captured when selected percentage was 10% (Best10) of lines by ranking of lines within each environment. Lines within each environment were ranked from most to least favorable. (4) Percentage of top-yielding lines captured when selected percentage was 20% of lines (Best20) by ranking of lines within each environment.

### Data availability

The R code along with data sets of Groundnut, Japonica, Indica and Maize are available at: https://github.com/osval78/Novel_LOEO_Model. While the remaining data sets are available at: https://data.cimmyt.org/dataset.xhtml?persistentId=hdl:11529/10548140.

## Results

Here, we provide the results in five subsections for four crops (Maize, Japonica, YET_1 and Indica) corresponding to four out of the eight data sets and the last one corresponding to a summary across traits and across the 8 datasets. The results for the rest of the datasets are provided in **Appendix A** and **B**. The results are reported in terms of APC, percentage of top-yielding lines captured when selected percentage was 10% (Best10) and 20% (Best20) of lines and in terms of normalized root mean square error (NRMSE).

### Data set 1. Maize

**Figure 1:**
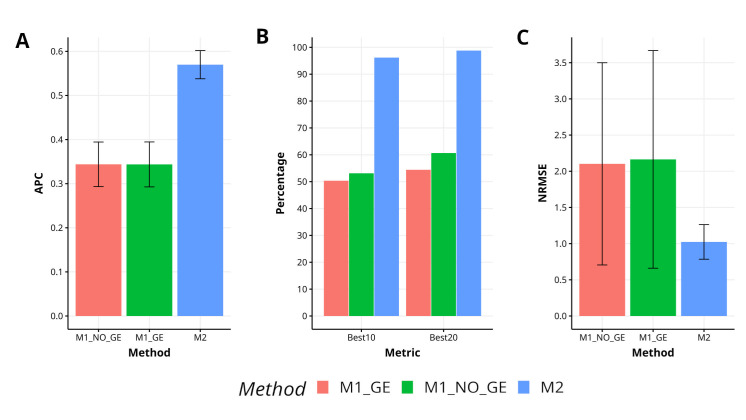
Results for data set 1 (Maize) in terms of **(A)** average Pearson´s correlation (APC), **(B)** percentage of top-yielding lines captured when selected percentage was 10% (Best10) and 20% (Best20) of lines and **(C)** normalized root mean square error (NRMSE).

In this data set, we can observe that the proposed method (M2) was better than M1_GE and M1_NO_GE methods in terms of APC by 65.8% and 65.7%, respectively. In terms of the power of the methods to capture the top best 10% of the lines (Best10), M2 is superior by 91.1% and 81.1%, regarding M1_GE and M1_NO_GE respectively, while in terms of the power of the methods to capture the top best 20% of the lines (Best20) M2 methods outperformed by 81.5% and 62.9% M1_GE and M1_NO_GE methods respectively. Regarding NRMSE method M2 was also superior in terms of prediction accuracy by 111.4% and 105.4% regarding M1_GE and M1_NO_GE respectively. In **Figure 1** can be observed that method M2 clearly outperforms M1 methods (M1_GE and M1_NO_GE). For more details see **Table A1** in **Appendix A**.

### Data set 2. Japonica

**Figure 2:**
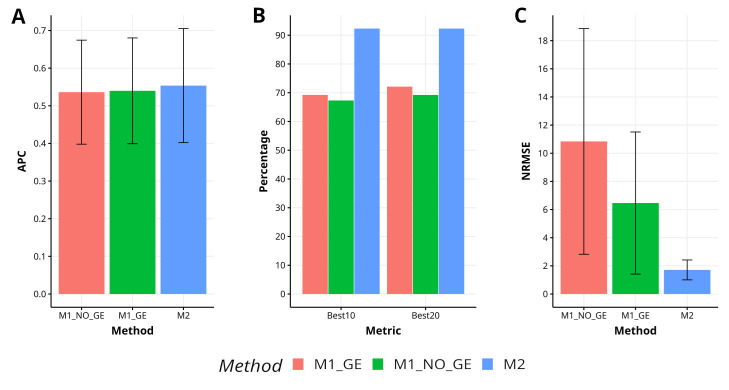
Results across traits for data set 2 (Japonica) in terms of **(A)** average Pearson´s correlation (APC), **(B)** percentage of top-yielding lines captured when selected percentage was 10% (Best10) and 20% (Best20) of lines and **(C)** normalized root mean square error (NRMSE).

In Japonica, the proposed method M2 has demonstrated a higher level of effectiveness in comparison to the methods M1_GE and M1_NO_GE, achieving a 2.6% and 3.2% increase in APC, respectively. The M2 method has also exhibited superior capability in capturing the top 10% and 20% of the lines (Best10 and Best20), with improvements of 33.3% and 37.1% over M1_GE and M1_NO_GE for Best10, and improvements of 28.0% and 33.3% over M1_GE and M1_NO_GE for Best20. Additionally, the M2 method has shown greater accuracy in predicting NRMSE, with improvements of 277.7% and 533.4% over M1_GE and M1_NO_GE, respectively. **Figure 2** visually represents the performance of M2 as superior to M1_GE and M1_NO_GE. For a more comprehensive analysis, refer to **Table A1** in **Appendix A**.

### Data set 3. EYT_1

**Figure 3:**
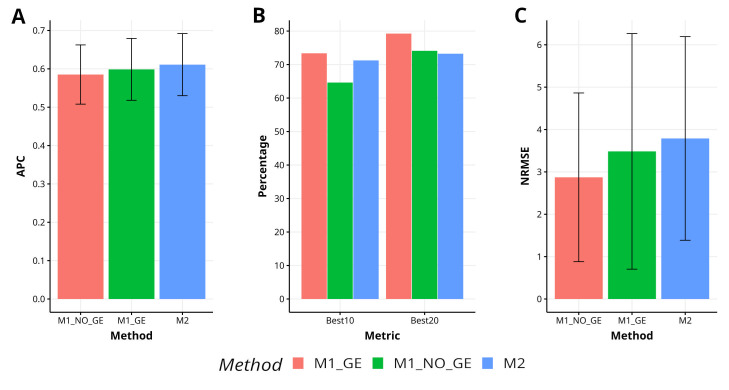
Results across traits for data set 3 (EYT_1) in terms of **(A)** average Pearson´s correlation (APC), **(B)** percentage of top-yielding lines captured when selected percentage was 10% (Best10) and 20% (Best20) of lines and **(C)** normalized root mean square error (NRMSE).

The effectiveness of the proposed method M2 in comparison to the methods M1_GE and M1_NO_GE has been evaluated in the YET_1 dataset, resulting in a 2.1% and 4.4% increase in APC, respectively. M2 has exhibited superior capability in capturing the top 10% of the lines (Best10), with an improvement of 10.2% over M1_NO_GE, but a decrease in improvement of 7.6% and 1.2% over M1_GE and M1_NO_GE for Best20, respectively. Additionally, M2 has shown a decrease in improvement in terms of NRMSE, with reductions of 8.1% and 24.8% over M1_GE and M1_NO_GE, respectively. **Figure 3** illustrates the performance of M2 in comparison to M1_GE and M1_NO_GE. For a more detailed analysis, refer to **Table A1** in **Appendix A**.

### Data set 4. Indica

**Figure 4:**
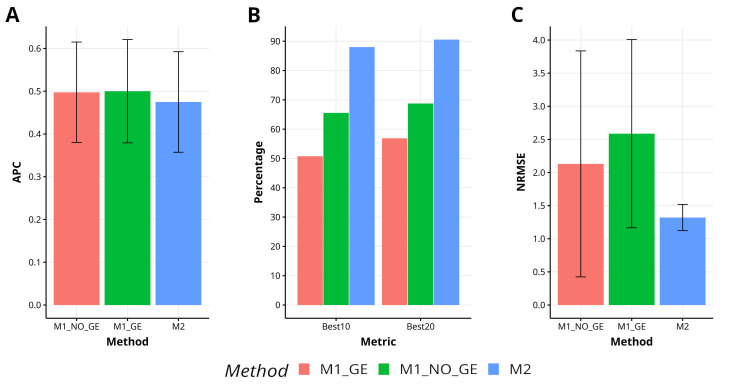
Results across traits for data set 6 (Indica) in terms of **(A)** average Pearson´s correlation (APC), **(B)** percentage of top-yielding lines captured when the selected percentage was 10% (Best10) and 20% (Best20) of lines and **(C)** normalized root mean square error (NRMSE).

In Indica, a comparison of the proposed method M2 with the methods M1_GE and M1_NO_GE has shown a lower level of effectiveness, resulting in a 5.0% and 4.5% decrease in APC, respectively. However, M2 has demonstrated superior capability in capturing the top 10% and 20% of the lines (Best10 and Best20), achieving improvements of 73.4% and 34.2% over M1_GE and M1_NO_GE for Best10, and improvements of 59.2% and 31.7% over M1_GE and M1_NO_GE for Best20. Additionally, M2 has exhibited greater accuracy in predicting NRMSE, with improvements of 95.9% and 61.4% over M1_GE and M1_NO_GE, respectively. **Figure 4** visually represents the superiority of M2 in comparison to M1_GE and M1_NO_GE. For a more detailed analysis, refer to **Table A1** in **Appendix A**.

### Across data sets

**Figure 5:**
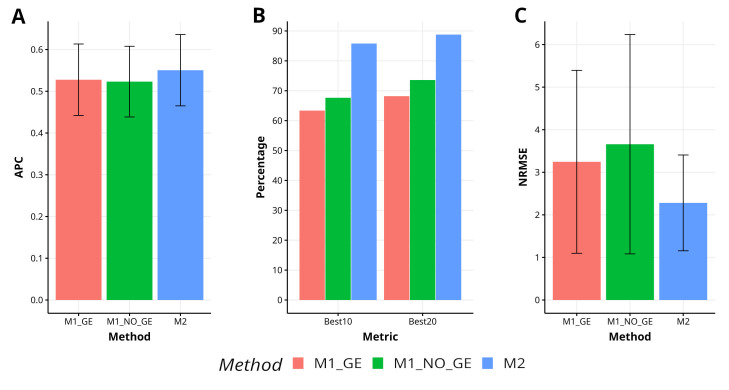
Results across traits and data sets for the two methods in terms of **(A)** average Pearson´s correlation (APC), **(B)** percentage of top-yielding lines captured when selected percentage was 10% (Best10) and 20% (Best20) of lines and **(C)** normalized root mean square error (NRMSE).

**Figure B1:**
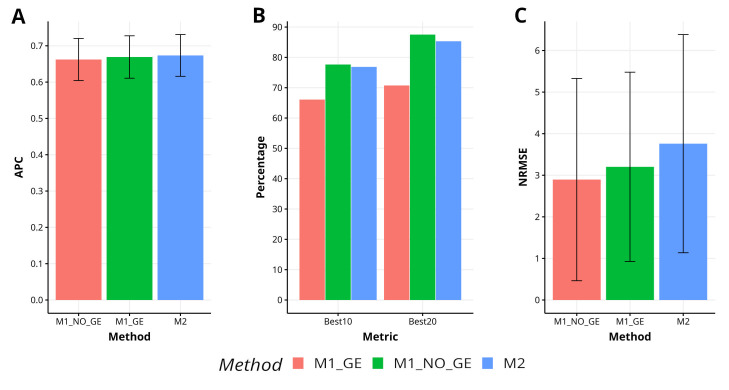
Results across traits for data set 4. EYT_2 in terms **(A)** average Pearson´s correlation (APC) and **(B)** percentage of top-yielding lines captured when selected percentage was 10% (Best10) and 20% (Best20) of lines and **(C)** normalized root mean square error (NRMSE).

**Figure B2:**
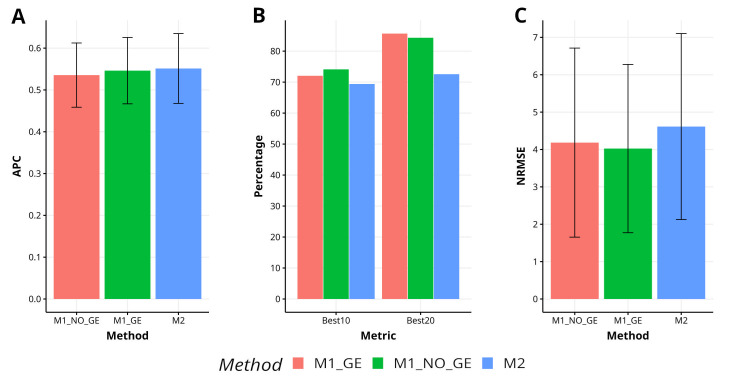
Results across trait for data set 5 (EYT_3) in terms of **(A)** average Pearson´s correlation (APC) and **(B)** percentage of top-yielding lines captured when selected percentage was 10% (Best10) and 20% (Best20) of lines and **(C)** normalized root mean square error (NRMSE).

**Figure B3:**
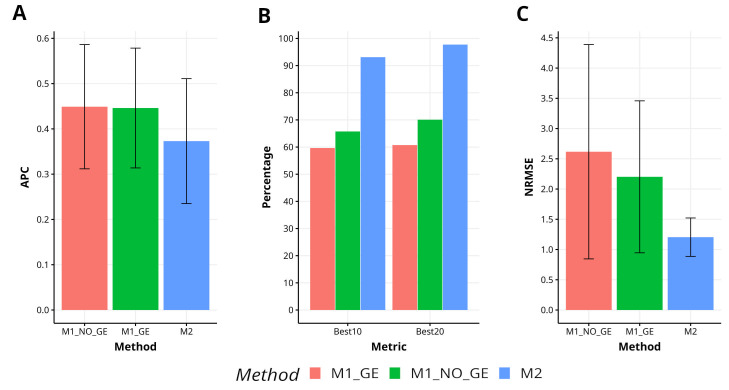
Results across trait for data set 7 (Groundnut) in terms **(A)** average Pearson´s correlation (APC) and **(B)** percentage of top-yielding lines captured when selected percentage was 10% (Best10) and 20% (Best20) of lines and **(C)** normalized root mean square error (NRMSE).

**Figure B4:**
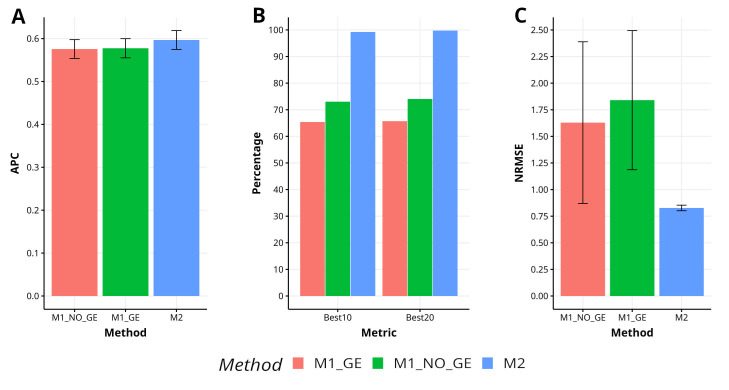
Results across trait for data set 8 (Disease) in terms of **(A)** average Pearson´s correlation (APC) and **(B)** percentage of top-yielding lines captured when selected percentage was 10% (Best10) and 20% (Best20) of lines and **(C)** normalized root mean square error (NRMSE).

Across various traits and data sets, it is evident that the proposed method (M2) outperforms the M1_GE and M1_NO_GE methods in terms of APC, with improvements of 4.3% and 4.5%, respectively. M2 has also shown superiority in capturing the top 10% of the lines (Best10), with improvements of 35.4% and 26.3% over M1_GE and M1_NO_GE, respectively. In terms of capturing the top 20% of the lines (Best20), M2 outperformed M1_GE and M1_NO_GE methods by 30.2% and 19.5%, respectively. Furthermore, M2 demonstrated superior prediction accuracy in terms of NRMSE, with improvements of 42.3% and 70.0% over M1_GE and M1_NO_GE, respectively. **Figure 5** visually depicts the superiority of M2 over the M1 methods (M1_GE and M1_NO_GE). For a more comprehensive analysis, please refer to **Table A1** in **Appendix A**. In the figures of **Appendix B** (**Figures B1**–**B4**) is summarized the prediction performance of methods M2, M1_GE and M1_NO_GE for the remaining data sets (EYT_2, EYT_3, Groundnut and Disease).

## Discussion

The primary goal of genomic prediction is to predict the genetic potential or performance of individuals for a trait of interest, such as yield, disease resistance, or quality traits. This prediction is based on the genetic markers that are present in the individual’s genome, which are used as predictors in a statistical model that relates them to the trait of interest.

However, in GS, the goal is to predict the performance of untested lines based on their genomic information. This prediction is typically based on a model that relates the genetic markers to the trait of interest using a training set of lines with known phenotypic values. The challenge is to ensure that the model is accurate and robust enough to predict the performance of untested lines, even though it has not seen its phenotypic data during training. This is because the genomic information of untested lines may differ from that of the training lines, due to genetic variation, environmental effects or other factors. Therefore, it is essential to develop robust and accurate models that can generalize well to new and diverse populations and thus achieve successful genomic selection.

The specific goals in genomic prediction may vary depending on the application and the breeding objectives. Some common goals include: (1) predicting tested lines in tested environments, (2) predicting untested lines in tested environments, (3) predicting tested lines in untested environments and (4) untested lines in untested environments. Of these four prediction goals, in general terms we believe that the ranking of the least to the most challenging is as follows (1)<(2)<(3)<(4). That is, goals (3) and (4) are more challenging since the impact of not having enough information of a new environment usually significantly increases the uncertainty of the predictions. This is related to the G×E interaction effect that influences the development and expression of an organism’s traits since it refers to the complex interplay between genetic factors and environmental factors and this G×E interaction effect cannot be estimated efficiently under prediction goals (3) and (4) due to lack of information of the new environment.

As pointed out before prediction goals (3) and (4) are very challenging since most of the time involve diverse environments and in order to improve predictions, it is common practice to enhance genomic prediction models with supplementary elements that consider the variability resulting from different environments and their interplay with the genotype (Widener et al., 2021). These enhanced GP models can be broadly classified into two primary categories: naïve or non-informed, and informed. The naïve or non-informed approach involves incorporating a primary random effect for the environment, along with a two-way interaction effect that considers the relationship between each marker genotype and each environment. The G × E model, also known as the genotype-environment interaction model, has demonstrated improved prediction accuracy compared to conventional GP models that only consider the main effects of genotype and environment (Jarquín et al., 2014; Lopez-Cruz et al., 2015; Widener et al., 2021).

The second approach, known as the informed approach, incorporates environmental covariates (ECs) that are measured in each environment. These ECs are then integrated into the model using kernel-based methods, allowing for the inclusion of variance-covariance structures that account for the interaction between environmental factors and marker genotypes. This resulting model incorporates the quantification of interactions between each marker genotype and each EC to predict GEBVs (genomic estimated breeding values). It has the potential to outperform the naïve G × E models (Jarquín et al., 2014; Basnet et al., 2019). However, still nowadays with these sophistication in the modeling process is very challenging to obtain high prediction accuracy in prediction goals (3) and (4) since: (a) it is not available the key information of the whole environment that need to be predicted and (b) there is a strong G × E interaction and mismatch between the training and testing distributions. For these reason novel approaches are required to improve prediction accuracy under these two prediction goals.

It is important to point out that the proposed method was designed for the prediction goal (3; tested lines in untested environments), and according to our results, it significantly outperforms the conventional methods (M1_GE and M1_NO_GE). For example, across traits and environments the proposed method (M2) outperformed the conventional method by at least 4.3% in terms of APC, while for capturing the best lines in the top 10% and 20% of the ordered lines in each environment there were gains of at least 26.3% and 19.5% respectively. However, in terms of NRMSE, the gain of the proposed M2 method was at least 42.3%. From these empirical results, we interpret that the proposed method significantly reduces the mismatch in distribution between the training and testing set and for this reason the proposed method improves the prediction accuracy of the lines in the new environment. Under this prediction goal, tested lines in untested environments and the mismatch in distribution between the training and testing set is attributed to non-stationary environments often caused by temporal or spatial changes that relate to the nature of the particular environment, which is very common in real-world applications in plant breeding programs. However, for the nature of the proposed M2 from our results we can infer that this method significantly reduces the mismatch in distribution between the training and testing sets.

We also believe that the proposed method is more efficient in terms of prediction power in part due the nature of how the final predictions are computed (See equation 3 in materials and methods), that is, the proposed method (M2) performs a peculiar type of ensemble that use the observed values of the tested lines on those environments that conform the training and the predicted differences corresponding to the testing. This means that the proposed method (M2) takes advantage of the virtues of ensemble methods which are: 1) improving the accuracy and robustness of machine learning models by combining the predictions of multiple models; 2) reducing the risk of overfitting and increasing the generalization ability of the final model, by leveraging the diversity of the models; 3) increasing the robustness of models by reducing the impact of outliers and errors in individual models; 4) being applied to a wide range of machine learning problems, including classification, regression, and clustering; and 5) handling large and complex datasets, as well as noisy and imbalanced data. For these reasons, ensemble methods are widely used in industry and academia, and have been shown to consistently improve the performance of machine learning models.

Although the proposed method enhances the accuracy of predictions when applied to tested lines in untested environments, predicting breeding values or phenotypic information for an entire environment using a reference population that has genotypic and phenotypic information from available environments is a challenging task. Typically, statistical machine learning methods struggle to attain good accuracy in this process. Therefore, research that can significantly improve prediction accuracy is crucial to ensuring the reliability of the GS methodology in breeding programs. Nevertheless, further empirical validations are necessary to ensure the effectiveness of the proposed method. Thus, we encourage other researchers to assess the proposed method using other data and the R code provided in https://github.com/osval78/Novel_LOEO_Model.

It should be noted that the proposed method can be implemented using various statistical machine learning algorithms. However, we chose to utilize the GBLUP model because it is currently one of the most potent and advanced models for GS. Additionally, this method offers the potential to incorporate other techniques, such as predictor markers and pedigree data. Nonetheless, further empirical evaluations are required to evaluate the efficacy of this method. Also, it is important to point out that the proposed method only was developed for tested lines in untested environments (new environments) for this reason additional work is required to apply this method to other cross-validation methods.

## Conclusions

In this research was proposed an alternative to improve the prediction accuracy under the scenario of predicting tested lines in untested environments. Instead of training the model with the original response variable available in the training set, the new method trained the model with a modified response variable (a difference of response variables) that helped to decrease the lack of a non-stationary distribution between the training and testing and as a by product improved the prediction accuracy. The gain in prediction accuracy of the proposed method, M2, regarding the conventional approach (M1_GE, M1_NO_GE) in terms of Pearson´s correlation was of at least 4.3%, while in terms of percentage of top-yielding lines captured when was selected the 10% (Best10) and 20% (Best20) of lines was at least of 19.5%, while in terms of NRMSE was of at least of 42.29%. These gains in prediction accuracy empirically show that the proposed method is very promising for genomic selection in the context of predicting tested lines in untested environments. For this reason, we encourage more empirical validation to support our findings.

## Data availability statement

The original contributions presented in the study are included in the article/supplementary material. Further inquiries can be directed to the corresponding authors.

## Author contributions

OM-L: Methodology, Investigations, Analyses, Writing. SR-P: Methodology, Writing. CH-S: Methodology, Writing. BM: Analyses, Writing. FV-A: Investigation, Writing. AM-L: Methodology, Investigations, Analyses, Writing. JC: Methodology, Writing. All authors contributed to the article and approved the submitted version.

## Appendix A

**Table A1:** Results of prediction accuracy for all datasets with the two methods in terms of NRMSE, APC, Best10 and Best20. RE_NRMSE was computed dividing the NRMSE of M1_GE (or M1_NO_GE) and the NRMSE of M2.

Dataset	Method	NRMSE	NRMSE_SE	APC	APC_SE	Best10	Best20	RE_NRMSE	RE_APC	RE_Best10	RE_Best20
Disease	M1_GE	1.8404	0.3335	0.5775	0.0114	65.3933	65.7133	2.224	1.033	1.518	1.518
Disease	M1_NO_GE	1.6291	0.3877	0.5756	0.0112	73.03	74.0333	1.969	1.037	1.359	1.347
Disease	M2	0.8274	0.0133	0.5967	0.0112	99.2367	99.7467				
EYT_1	M1_GE	3.484	1.4185	0.5986	0.0411	73.3675	79.2475	0.919	1.021	0.971	0.924
EYT_1	M1_NO_GE	2.872	1.0158	0.5852	0.0394	64.625	74.1025	0.758	1.044	1.102	0.988
EYT_1	M2	3.7893	1.2261	0.611	0.0413	71.24	73.2425				
EYT_2	M1_GE	3.2028	1.1613	0.6691	0.0298	66.085	70.74	0.852	1.006	1.163	1.206
EYT_2	M1_NO_GE	2.896	1.2411	0.6622	0.0296	77.6475	87.515	0.77	1.017	0.99	0.975
EYT_2	M2	3.7597	1.3385	0.6734	0.0293	76.8725	85.32				
EYT_3	M1_GE	4.0245	1.1487	0.5462	0.0405	72.0425	85.66	0.872	1.009	0.963	0.847
EYT_3	M1_NO_GE	4.1838	1.2904	0.5355	0.0392	74.1175	84.31	0.907	1.03	0.936	0.861
EYT_3	M2	4.6132	1.2692	0.5513	0.0426	69.3975	72.56				
Groundnut	M1_GE	2.2021	0.641	0.4461	0.0675	59.6475	60.73	1.829	0.836	1.561	1.609
Groundnut	M1_NO_GE	2.6168	0.9039	0.449	0.07	65.7475	70.0775	2.173	0.831	1.416	1.395
Groundnut	M2	1.2043	0.1619	0.373	0.0704	93.1075	97.7375				
Indica	M1_GE	2.5865	0.7248	0.5	0.0616	50.765	56.8875	1.959	0.95	1.734	1.592
Indica	M1_NO_GE	2.1303	0.8705	0.4974	0.06	65.56	68.75	1.614	0.955	1.342	1.317
Indica	M2	1.3202	0.1009	0.4748	0.06	88.01	90.565				
Japonica	M1_GE	6.4636	2.5752	0.5398	0.0717	69.2325	72.1175	3.777	1.026	1.333	1.28
Japonica	M1_NO_GE	10.8401	4.0901	0.5362	0.0705	67.3075	69.2325	6.334	1.032	1.371	1.333
Japonica	M2	1.7115	0.3607	0.5536	0.0773	92.3075	92.3075				
Maize	M1_GE	2.1638	0.7671	0.3437	0.026	50.35	54.42	2.114	1.658	1.91	1.815
Maize	M1_NO_GE	2.1023	0.7128	0.3439	0.0258	53.12	60.66	2.054	1.657	1.811	1.629
Maize	M2	1.0235	0.1219	0.5698	0.0163	96.18	98.79				
Across_data	M1_GE	3.246	1.0963	0.5276	0.0437	63.3604	68.1895	1.423	1.043	1.354	1.302
Across_data	M1_NO_GE	3.8772	1.3774	0.5268	0.0408	67.9421	74.2758	1.7	1.045	1.263	1.195
Across_data	M2	2.2811	0.5741	0.5505	0.0436	85.794	88.7837				

While the RE_APC, RE_Best10, RE_Best20 were computed dividing the APC, Best10 and Best20 of M2 model and the APC, Best10 and Best20 of M1_GE (or M1_NO_GE).
